# Chemical Oxidation of Chrysene: A Structural and Theoretical Description of a Mixed-Valent Trimeric Radical Cation

**DOI:** 10.1021/acs.cgd.5c01176

**Published:** 2025-11-10

**Authors:** Megan E. McCormack, Rameswar Bhattacharjee, Zheng Wei, Marina A. Petrukhina

**Affiliations:** † Department of Chemistry, 1084 University at Albany, State University of New York, Albany, New York 12222, United States; ‡ Department of Chemistry and Institute of Soft Matter, 8368 Georgetown University, 37th and O Streets, NW, Washington, D.C. 20057-1227, United States

## Abstract

Chemical oxidation of chrysene with GaCl_3_ affords a trimeric chrysene radical dication, [(C_18_H_12_)_3_]^2+^, crystallized with two Ga_2_Cl_7_^–^ anions, as revealed by single crystal X-ray diffraction analysis. In the crystal structure, 1D π-stacked columns of chrysene with interplanar contacts averaged at 3.303(3) Å are separated by Ga_2_Cl_7_^–^ anions. Computational studies reveal that the positive charge is concentrated largely on the terminal chrysene units with charges of +0.84 and +0.80, while the center chrysene has a comparatively lower charge of +0.36. This charge distribution is corroborated with structural features of the trimer, as the terminal chrysene molecules exhibit a more pronounced core deformation. Taking the neutral trimer as a reference, much weaker interactions are observed in the title chrysene trimer bearing a +2 charge, suggesting the presence of substantial electrostatic repulsion.

## Graphical abstract

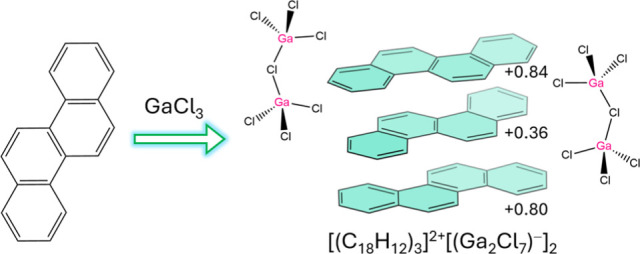

Polycyclic aromatic hydrocarbons (PAHs) with variable size and symmetry continue to attract significant attention due to their interesting optical and electrical properties.^1^ Partial oxidation of PAHs to form the radical cation π-stacked aggregates is used for tuning electronic and conductive properties of the resulting solid-state materials.^2^ Upon oxidation, the radical cations of PAHs can generate mixed-valent dimers^3,4^ or trimers in the solid-state.^5,6^ The resulting open-shell π-stacked products with electron transport pathways find applications in organic electronics, solar cells and batteries.^7^ However, the functionality of the conductive pathways is largely dependent on the particular solid-state configuration that could facilitate intermolecular charge transfer.^8^ Therefore, modification of the solid-state packing arrangement from the typical herringbone structures of neutral PAHs to the continuous π-stacks is essential for accessing new efficient materials for organic electronics.^9^

One planar PAH in particular, chrysene (C_18_H_12_), is known to exhibit blue fluorescence, with multiple π-expanded and functionalized derivatives synthesized for applications in organic light-emitting diodes (OLEDs).^10^ Furthermore, doping chrysene with potassium and formation of charge transfer complexes with TCNQ reveal its superconducting properties at low temperatures.^11,12^ However, the redox properties of chrysene have been sparsely investigated. Only in two reports on the chemical oxidation with SbCl_5_ or SbF_5_ in CH_2_Cl_2_ (DCM) or SO_2_ClF, respectively, was the formation of the radical cation detected in solution through *in situ* EPR^13^ and NMR spectroscopy.^14^ Beyond these examples, generation of chrysene radical cations is limited to ionization through UV-irradiation^15^ and γ-irradiation for *in situ* measurements of the electronic spectra, vibronic absorption and IR spectra.^16–18^ The cation radical of chrysene was also generated by hydrogen abstraction through sustained off-resonance irradiation collision-induced dissociation (SORI-CID) and characterized by FT-ICR mass spectroscopy.^19^ Several cationic transition metal complexes with chrysene can be mentioned here,^20–23^ but so far, no radical cations of chrysene have been successfully isolated in the crystalline form, thus precluding further investigation.

The limited studies of chrysene oxidation also hamper theoretical analysis of bonding in the resulting π-stacked systems. So far, bonding descriptions of dimeric^24–26^ and trimeric^5,27^ cation radicals have been provided for such planar PAHs as perylene^25^ and triphenylene.^5^ The theory helps to accurately describe orbital interactions within π-column arrangements as well as to detect pancake bonding interactions between the positively charged small PAHs.^28^ Crystallographically characterized cationic PAH-based radical systems often exhibit structural features associated with pancake bonding interactions, with interplanar contacts below 3.4 Å and a slightly offset overlap to impede electrostatic repulsions.^29^ However, to get deep insights into pancake bonding, help from density functional theory (DFT) calculations is needed.

To address the lack of controlled chemical oxidation methods, the reaction of chrysene with the mild oxidant GaCl_3_ (0.40 eV) was tested under ambient conditions. This reaction successfully afforded a new chrysene radical cation, which was characterized through single crystal X-ray diffraction, EPR, UV–vis spectroscopic methods, ATR-IR spectroscopy, and conductivity measurements. Additionally, computational studies are employed to further evaluate bonding interactions and charge distribution in the resulting product.

One-pot mixing of chrysene and GaCl_3_ in anhydrous fluorobenzene at room temperature immediately afforded a deep fuchsia solution. Crystals suitable for X-ray diffraction analysis were grown through cooling of the reaction mixture at 5 °C. After several days, dark purple needles were deposited in a moderate yield. X-ray diffraction analysis confirmed the product composition as [(C_18_H_12_)_3_]^2+^[(Ga_2_Cl_7_)^−^]_2_ (Figure 1). Crystals conform to a monoclinic *P*2_1_/*c* space group (*Z* = 2), with a volume of 2756.38(5) Å^3^.

**Figure 1:**
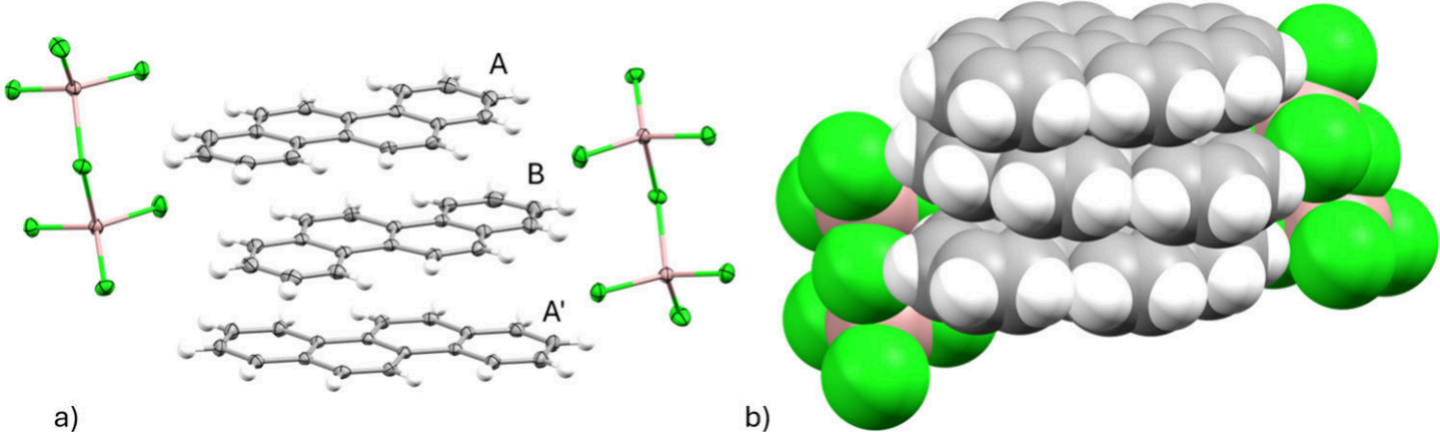
(a) ORTEP drawing of [(C_18_H_12_)_3_]^2+^[(Ga_2_Cl_7_)^−^]_2_ with thermal ellipsoids shown at the 50% probability level and (b) space-filling model of [(C_18_H_12_)_3_]^2+^[(Ga_2_Cl_7_)^−^]_2_.

The asymmetric unit contains 1.5 chrysene per Ga_2_Cl_7_^–^. The trimeric chrysene unit with two Ga_2_Cl_7_^–^ anions is built through an inversion center on the central chrysene B, with chrysene A and A′ being crystallographically equivalent (Figure 1a, S9). Within the crystal structure, continuous stacks of chrysene are formed down the *a*-axis, and each π-stack is charge-separated by anionic columns (Figure S8). Multiple short contacts between chrysene range over 3.137(3)–3.387(3) Å, comprising 1D columns with no distinct trimeric units evident based on crystallographic analysis (Figures 2a, S10). Within trimers, a shifted ring-over-atom overlap is observed, while the top and bottom chrysene of neighboring trimers show an atom-over-atom overlap (Figure 2b). The intercolumn interactions are comprised of H···Cl contacts. While chrysene A and A′ exhibit one H···Cl contact with an adjacent Ga_2_Cl_7_^–^ anion (2.890(6) Å), the central chrysene B shows two H···Cl contacts with different anions, both at 2.892(6) Å (Figure 2c).

**Figure 2:**
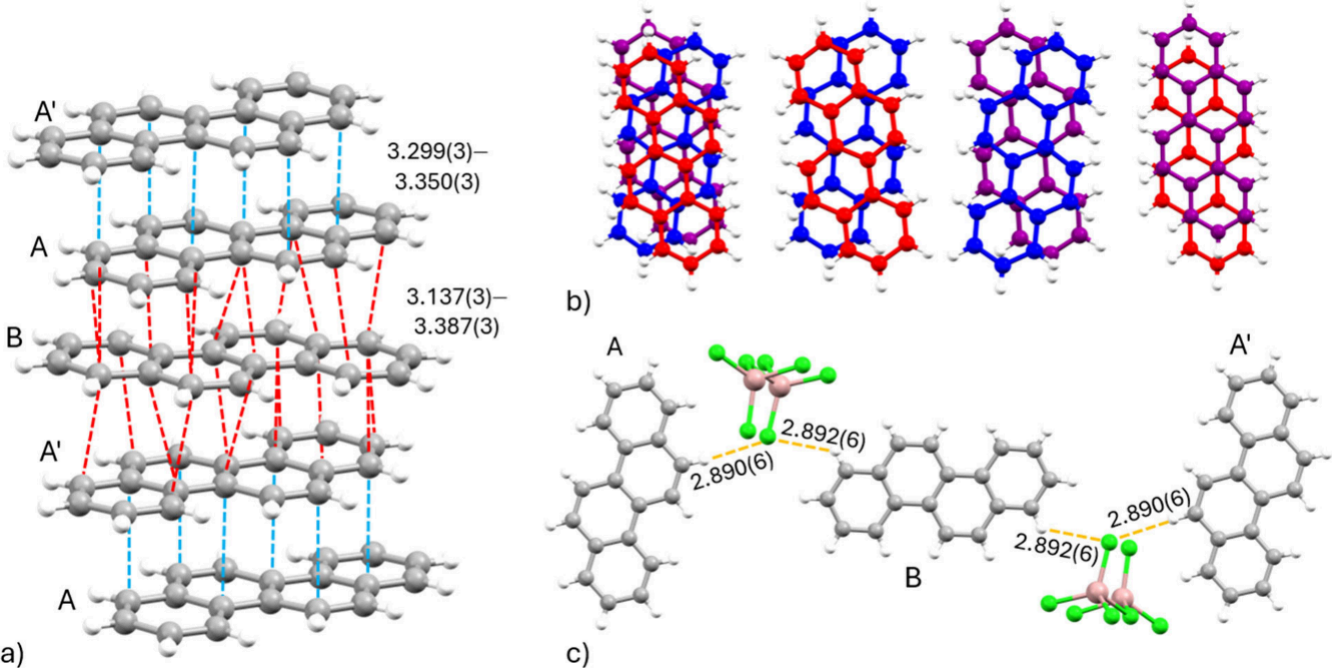
(a) Shortest interplanar contacts (Å) in [(C_18_H_12_)_3_]^2+^[(Ga_2_Cl_7_)^−^]_2_. (b) Overlap modes within trimeric units of chrysene: chrysene A is red, chrysene B is blue, and chrysene A′ is purple. (c) H···Cl interactions (Å) in [(C_18_H_12_)_3_]^2+^[(Ga_2_Cl_7_)^−^]_2_.

The minor core deformation of the oxidized chrysene within each trimeric unit can be seen from the differences in the C–C bond length and changes in dihedral angle compared to neutral parent^30^ (Table S3). In chrysene B, bond length contraction (0.013–0.018 Å) is observed in bonds a and k, while bond length elongation (0.013–0.027 Å) is detected in bonds c and d. For chrysene A (and A′), only five bonds experience notable elongation (c, f, and i) or contraction (a and k). Compared to the neutral chrysene, there is a notable increase in dihedral angles between rings A and D from 0° to 4.34° in the oxidized product, indicating chrysene A experiences an overall decrease in planarity (Figure 3). These structural features suggest some charge delocalization throughout the trimeric unit.

**Figure 3:**
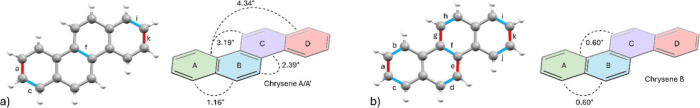
Dihedral angles (deg) and C–C bond elongation (shown in blue) and contraction (shown in red) (Å) in (a) chrysene A and A′ and (b) chrysene B.

Additional product characterization was performed through EPR, UV–vis and ATR-IR spectroscopy, powder X-ray diffraction, and conductivity measurements. The EPR spectrum (Figure S1), collected on crystals of [(C_18_H_12_)_3_]^2+^[(Ga_2_Cl_7_)^−^]_2_ packed under argon, revealed a strong singlet with a *g*-factor of 2.0048, indicative of a delocalized organic radical. The EPR spectra were subsequently collected in the temperature range of +80 to −80 °C (Figure S2). As the temperature decreased, the signal intensity decreased slightly; however, no major changes were observed. Moreover, powder X-ray diffraction analysis performed at variable temperatures (100, 233.15, 253.15, 273.15, and 293.15 K) confirmed the phase purity of the bulk crystalline product (Figure S6, Table S1) and revealed no phase transitions in the broad temperature range (Figure S7).

A comparison of the UV–vis absorption spectrum of the [(C_18_H_12_)_3_]^2+^[(Ga_2_Cl_7_)^−^]_2_ crystals dissolved in fluorobenzene (Figure S3) to the solution of neutral chrysene in fluorobenzene shows a small peak at 540 nm. Increasing product concentration more clearly defines the peak at 540 nm, which is not present in the neat parent and can be assigned to the positively charged chrysene. Diffuse UV–vis reflectance (Figure S4) was used to further evaluate the stability of the crystalline product, which exhibited near-immediate decomposition when exposed to air. Regarding ATR-IR spectroscopy, a similar pattern is observed in crystalline [(C_18_H_12_)_3_]^2+^[(Ga_2_Cl_7_)^−^]_2_ compared to a monomeric chrysene cation prepared through *in situ* vacuum ultraviolet irradiation.^18^ The collected spectrum (Figure S5) exhibits peaks in the 1200–1600 cm^–1^ and 750–850 cm^–1^ ranges with slight increases and decreases in intensity, respectively, relative to neutral chrysene. The conductivity measured on a pressed pellet of the crystalline [(C_18_H_12_)_3_]^2+^[(Ga_2_Cl_7_)^−^]_2_ sample at room temperature is 0.00023 S/cm. This value is notably lower compared to the chemically oxidized dimeric perylene cation, [(C_20_H_12_)_2_]^•+^(SbCl_6_^–^) (0.0055 S/cm),^31^ and several electrochemically oxidized planar PAHs, such as naphthalene, fluoranthene,^32^ and coronene^4,6,33^ (from 0.04 S/cm to 3.0 S/cm). However, the measured conductivity of [(C_18_H_12_)_3_]^2+^[(Ga_2_Cl_7_)^−^]_2_ shows a significant increase from that for the parent neutral chrysene (2 × 10^–19^ S/cm).^34^

To gain insight into the product structure and electronic properties, further characterization of the system was carried out by using DFT calculations. For this purpose, the crystallographically equivalent trimeric chrysene radical dication was isolated and optimized using DFT (see ESI for more details). The resulting structure, shown in Figure 4a, corresponds to an open-shell singlet in the ground state.

**Figure 4:**
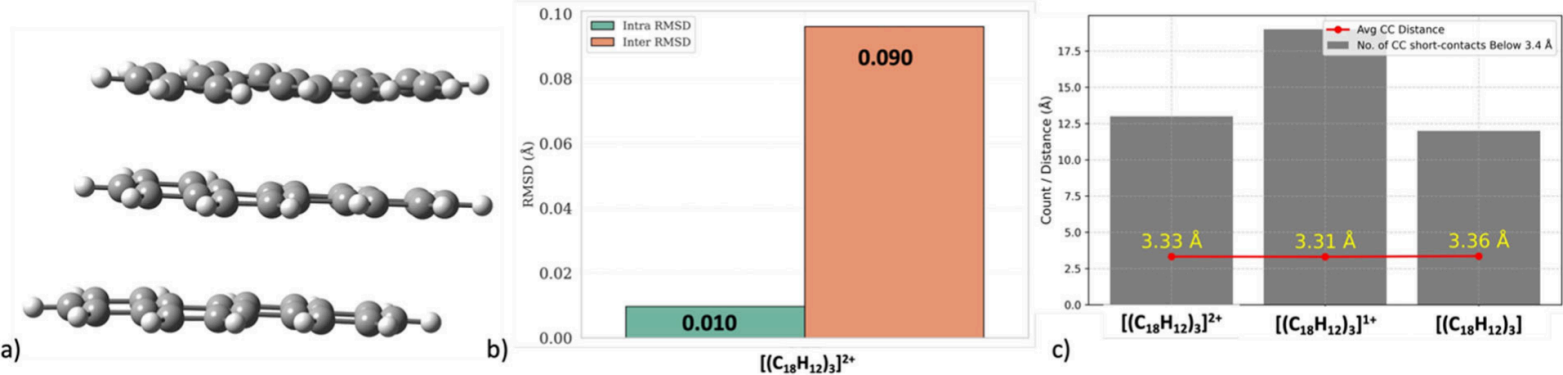
(a) DFT-optimized structure of trimeric radical dication [(C_18_H_12_)_3_]^2+^. (b) Root mean square deviation (RMSD) between the optimized structure and its crystallographic counterpart, separated into intra- and intermolecular components. (c) Number of intermolecular C···C short contacts (*m* in eq 3) and average interplanar distances (*d*_av_) in [(C_18_H_12_)_3_]^*n*^ as a function of total charge (*n* = +2, +1, 0).

Agreement between the DFT-optimized geometry and the experimental crystal structure was assessed by calculating the root-mean-square deviation (RMSD) using eq 1. RMSD was further divided into two components: intra-RMSD, which includes only intramolecular C–C bonds, and inter-RMSD, which accounts for intermolecular C···C short contacts (Figure 4b). As expected, the intra-RMSD is very small (∼0.01 Å), indicating that the chosen level of theory accurately reproduces the molecular geometry. The inter-RMSD is slightly higher (∼0.09 Å) but still in good agreement, especially considering the greater translational freedom of the monomeric units in the gas-phase optimization.

The [(C_18_H_12_)_3_]^2+^ trimer contains 13 intermolecular C···C short contacts (defined as contacts shorter than the van der Waals cutoff of 3.4 Å) with an average intermonomer distance of 3.33 Å (Figure 4c). To examine the influence of charge on the packing behavior, the trimer was further optimized in the +1 and neutral charge states. Upon reducing the total charge from +2 to +1, the number of short C···C contacts increases to 18, and the average intermonomer distance (*d*_av_) decreases slightly to 3.31 Å. Although the +1-charged trimer exhibits interesting structural characteristics, a detailed discussion is beyond the scope of this communication. In the neutral state, the interplanar distance (*d*_av_) increased to 3.36 Å. These results suggest that increasing the positive charge generally leads to tighter packing, likely due to enhanced pancake interaction.^35^ However, at higher charge states, the resulting electrostatic repulsion may partially offset this effect, introducing a subtle balance between attractive and repulsive forces in determining the final packing arrangement. Notably, the neutral trimer of chrysene is a computed structure, and crystallographic studies of neutral chrysene reveal a herringbone-like packing motif with no π-stacking interactions.^30^

1 $$\mathrm{RMSD}=\frac{1}{N}\sqrt{\overset{N}{\underset{i=1}{\sum }}{\left(d_{i}^{\mathrm{crystal}}-d_{i}^{\mathrm{DFT}}\right)}^{2}}$$

2 $$E_{\mathrm{int},b}=E_{\mathrm{trimer}}^{+2}-2\times E_{\mathrm{chrysene}}^{+1}-E_{\mathrm{chrysene}}^{\mathrm{neutral}}$$

The interaction energy between the chrysene units in the dipositive trimer is calculated to be relatively weak (−2.0 kcal/mol, using eq 2), whereas the interaction energy in the neutral trimer with the same geometry is significantly stronger, at −21.9 kcal/mol. Taking the neutral trimer is taken as a reference for van der Waals-driven interactions, the much weaker interaction observed in the chrysene trimer bearing +2 charge suggests the presence of substantial electrostatic repulsion.

To better understand this repulsion, we analyzed the charge distribution within the trimer using CHELPG population analysis.^36^ Summation of atomic charges across each monomer reveals that the terminal chrysenes bear the majority of the +2 charge, while the central chrysene is comparatively less positively charged. As shown in Figure 5a, chrysene A carries +0.84 charge and chrysene A′ carries +0.80, leaving only +0.36 on the central chrysene B. Since all three monomers are positively charged, electrostatic repulsion plays a critical role in the trimer’s energy landscape. To quantify this effect, we calculated pairwise Coulombic repulsion energies between adjacent monomers using CHELPG-derived atomic charges. The repulsion between chrysene A and B is estimated to be 14.4 kcal/mol, while that between chrysene B and A′ is 13.9 kcal/mol, resulting in a total Coulombic repulsion of 28.3 kcal/mol for the trimer. Notably, the asymmetric charge distribution appears to stabilize the trimer by placing the most highly charged monomers at opposite ends, thereby minimizing their direct electrostatic repulsion. This trend is corroborated by the spin density map shown in Figure 5b, which reveals that spin density is primarily localized on the terminal chrysenes, with minimal contribution from the central unit. Furthermore, to probe the nature of intermonomer electronic communication, we examined four sets of occupied and one set of unoccupied valence orbitals (Figure S11). These orbitals exhibit intermonomer overlap characteristic of pancake bonding.^35^ However, identifying a definitive pair of bonding and antibonding orbitals responsible for such interactions proved to be challenging, making it difficult to assign a formal bond order to the pancake bond in this system.

**Figure 5:**
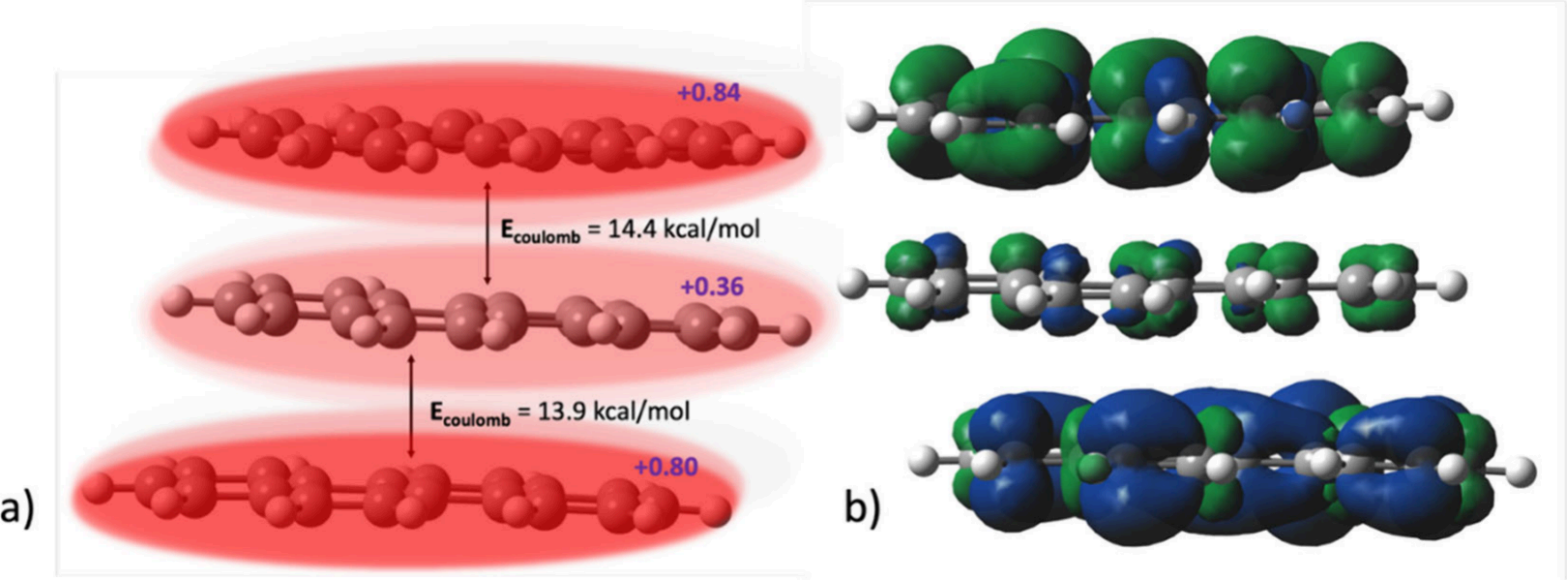
(a) Distribution of the +2 charge across the three monomer units in the [(C_18_H_12_)_3_]^2+^ trimer, obtained from CHELPG population analysis. (b) Spin density map of [(C_18_H_12_)_3_]^2+^ showing localization primarily on the terminal chrysenes.

In summary, the utility and the versatility of GaCl_3_ as an effective oxidant for partial oxidation of PAHs is further demonstrated, expanding the toolbox of synthetic and structural chemists.^37^ Specifically, a chrysene radical cation product was obtained through chemical oxidation with GaCl_3_ and crystallized as [(C_18_H_12_)_3_]^2+^[(Ga_2_Cl_7_)^−^]_2_. The X-ray diffraction analysis revealed that the product contains 1D π-stacks of positively charged chrysene units charge-separated by the dimeric gallium­(III) halide anions, Ga_2_Cl_7_^–^. The interplanar distances within the trimers (3.137(3)–3.387(3) Å) and the π-stacks (3.292(3)–3.392(3) Å) do not allow to clearly differentiate individual units in the extended 1D columns. The radical nature of the product was confirmed by EPR spectroscopy, while UV–vis spectroscopy indicated some charge delocalization through the cationic chrysene units. Complementary DFT calculations revealed that the terminal chrysenes in the trimer bear most of the positive charge, while the central chrysene retains a comparatively lower charge. Spin density plots also corroborate this delocalization, illustrating a radical character distribution across all the chrysene units but with primarily localization on the terminal chrysenes.

Further development of efficient chemical oxidation methods with control over charge and solid-state structures is important for the preparation of new organic-radical-based products to advance their electronic and optoelectronic applications.

## Supplementary Material



## Notes

Experimental and computational details, UV–vis spectra, EPR spectra, ATR-IR spectrum, conductivity sample preparation details, powder and single crystal X-ray diffraction data (PDF)
