# Supplementary material for: Chemical Oxidation of Chrysene: A Structural and Theoretical Description of a Mixed-Valent Trimeric Radical Cation
Source: Cryst Growth Des. 2025 Nov 10;25(22):9549–54. doi: 10.1021/acs.cgd.5c01176 (PMC12635959; doi:10.1021/acs.cgd.5c01176)
Supplement: Supplementary file 1 [file cg5c01176_si_001.pdf]

Supporting Information

Chemical Oxidation of Chrysene:
A Structural and Theoretical Description of a Mixed-Valent Trimeric Radical Cation

Megan E. McCormack,[1‡] Rameswar Bhattacharjee,[2‡] Zheng Wei,[1] Marina A. Petrukhina[1,*]

[1]Department of Chemistry, University at Albany, State University of New York, Albany, New York 12222, USA

[2]Department of Chemistry and Institute of Soft Matter, Georgetown University, 37th and O Streets, NW, Washington, DC 20057-1227, USA

*email: mpetrukhina@albany.edu

Table of Contents

I. Materials and Methods 3
II. Characterization of $[(C_{18}H_{12})_3]^{2+}[(Ga_2Cl_7)^{-}]_2$ 4
III. Crystal Structure Solution and Refinement 12
IV. Computational Details 17
V. References 27

I. Materials and Methods

All manipulations were carried out using break-and-seal[1] and glove-box techniques under an atmosphere of argon. Fluorobenzene (99%) was dried over 4 Å molecular sieves and degassed. Chrysene (≥98.0%) was purchased from TCI America and purified through sublimation at 200 °C prior to use. Gallium(III) chloride (ultra dry ≥99.999%) was purchased from Thermo Scientific and used as received. The UV-vis absorption spectra were recorded on a Shimadzu 2600i UV visible Spectrophotometer. The EPR spectrum was recorded on a LINEV ADANI Spinscan X Electron Paramagnetic Resonance Spectrometer. The UV-Vis diffuse reflectance spectra were recorded on a Jasco V-770 Spectrophotometer. The IR spectrum was collected on a Shimadzu IRTracer-100 Fourier Transform Infrared Spectrometer QATR10 Single Reflection ATR accessory. Powder X-ray diffraction data were collected on a Rigaku XtaLAB Synergy-S diffractometer equipped with a microfocus Cu Kα radiation source (λ = 1.54184 Å). The measurements were performed sequentially at 100 K (−173.15 °C), 233.15 K (−40 °C), 253.15 K (−20 °C), 273.15 K (0 °C), and 293.15 K (20 °C) with a temperature-ramp rate of 0.5 K min^{-1}.

$[(C_{18}H_{12})_3]^{2+}[(Ga_2Cl_7)^-]_2$

Fluorobenzene (1.5 mL) was added to a custom-built glass system[2] containing chrysene (5.0 mg, 0.0219 mmol). To the stirring suspension, $GaCl_3$ (5.7 mg, 0.0329 mmol) was added under inert atmosphere. The mixture immediately turned to a fuchsia color and was stirred at room temperature for 15 minutes. The reaction mixture was then filtered to afford the fuchsia solution. The ampule was sealed under reduced pressure and left at 5 °C to produce dark purple needles in 5 days. Yield: 3.6 mg, 34.2%. ATR-IR: 3051, 1612, 1514, 1483, 1423, 1263, 1192, 1139, 862, 813, 752, 576, 418 cm^{-1}.

II. Characterization of $[(C_{18}H_{12})_3]^{2+}[(Ga_2Cl_7)^-]_2$

EPR Spectroscopic Investigation

Sample Preparation: Crystals of $[(C_{18}H_{12})_3]^{2+}[(Ga_2Cl_7)^-]_2$ were transferred into the glovebox where solvent was removed. The crystals were dried *in-vacuo*. The dark needles (2.0 mg) were loaded into a quartz capillary tube (O.D. 1.25 mm) under argon. The tube was sealed, and the EPR spectrum was collected at 29.8 °C.

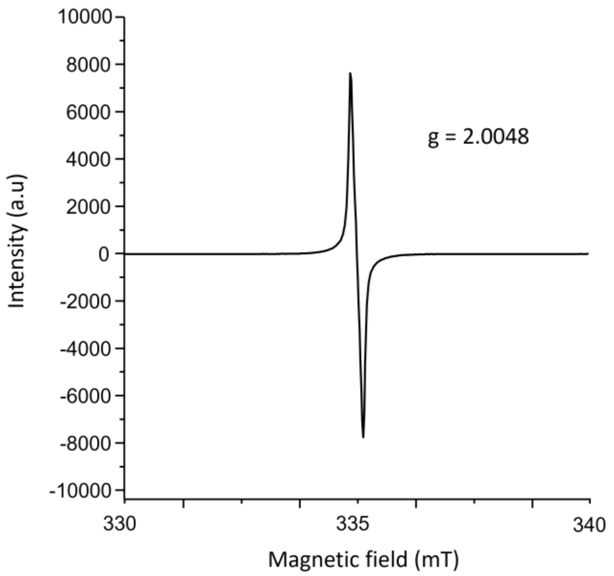

Figure S1. EPR spectrum of crystalline $[(C_{18}H_{12})_3]^{2+}[(Ga_2Cl_7)^-]_2$.

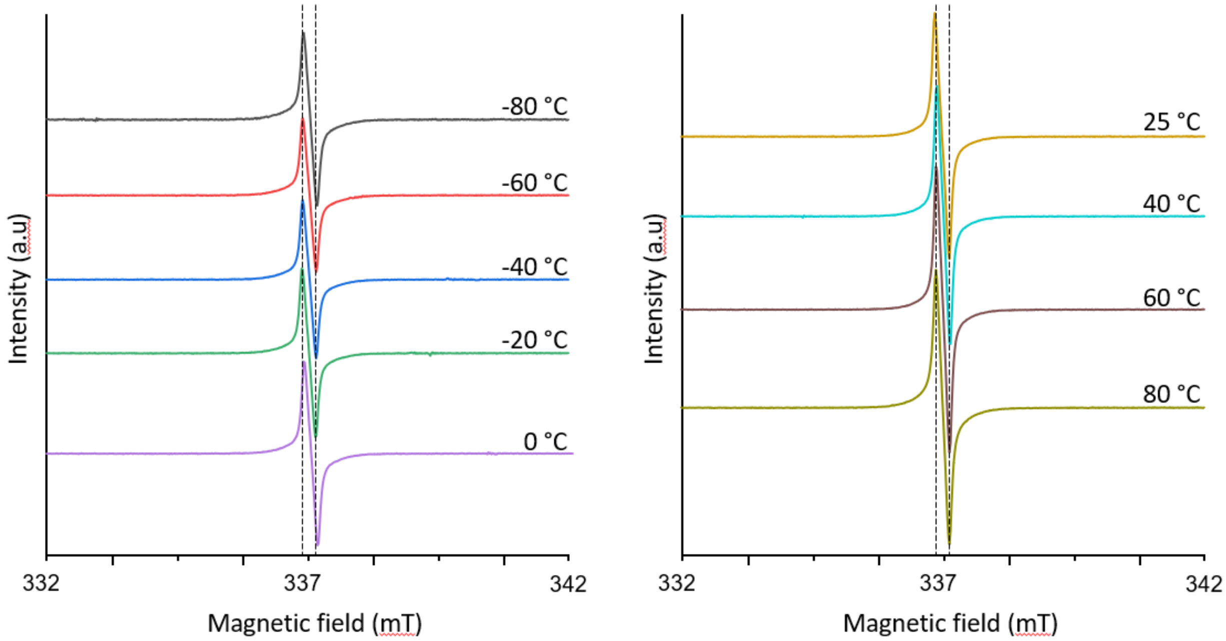

Figure S2. Variable temperature EPR spectra of crystalline $[(C_{18}H_{12})_3]^{2+}[(Ga_2Cl_7)^{-}]_2$, collected at -80 °C, -60 °C, -40 °C, -20 °C, and 0 °C (left) and 25 °C, 40 °C, 60 °C, and 80 °C (right) under Ar atmosphere.

UV-Vis Spectroscopic Investigation

Sample preparation: Several dark purple crystals of $[(C_{18}H_{12})_3]^{2+}[(Ga_2Cl_7)^{-}]_2$ were dissolved in fluorobenzene (1.5 mL). Separately, chrysene (0.2 mg) was dissolved in fluorobenzene (1.5 mL). The ampules were sealed under argon and the UV-Vis absorption spectra were recorded at 25 °C.

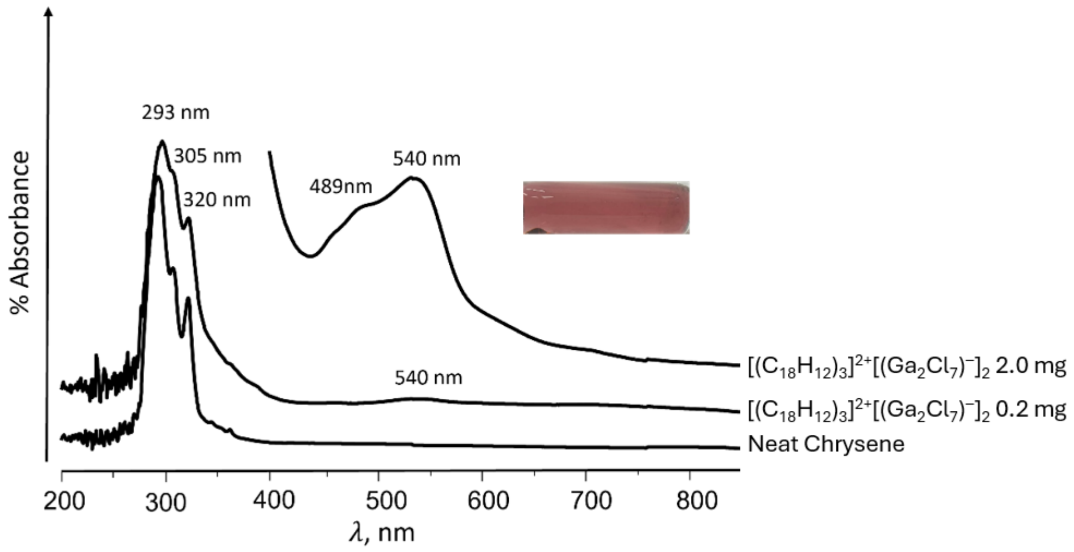

Figure S3. UV-Vis absorption spectra of $[(C_{18}H_{12})_3]^{2+}[(Ga_2Cl_7)^{-}]_2$ crystals dissolved in fluorobenzene *vs*. neat chrysene in fluorobenzene.

Sample preparation: Crystals of $[(C_{18}H_{12})_3]^{2+}[(Ga_2Cl_7)^{-}]_2$ were transferred into the glovebox where the purple solution was removed. The crystalline material (6.0 mg) was dried *in-vacuo* and loaded into the sample holder under argon. The solid-state UV-Vis diffuse reflectance spectra were monitored up to 130 minutes.

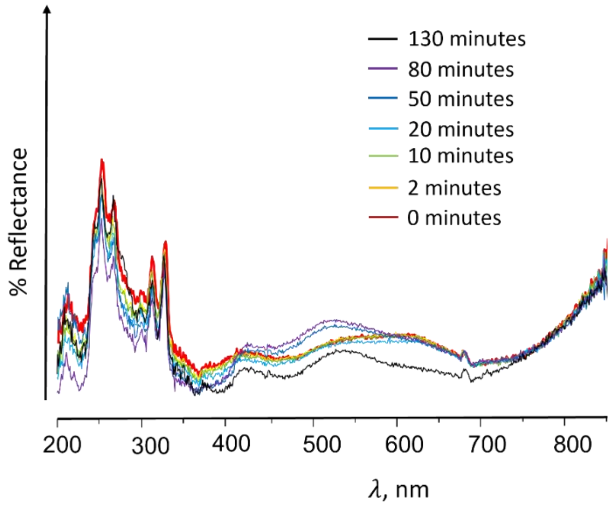

Figure S4. Diffuse reflectance spectra of crystalline $[(C_{18}H_{12})_3]^{2+}[(Ga_2Cl_7)^{-}]_2$ over 130 minutes. Spectra were collected on a Jasco V-770 Spectrophotometer.

ATR-IR Spectroscopic Investigation

Sample Preparation: Crystals of $[(C_{18}H_{12})_3]^{2+}[(Ga_2Cl_7)^-]_2$ were transferred into the glovebox where solvent was removed. The crystals (4.0 mg) were dried *in-vacuo* and loaded into the sample holder under argon. The sample holder was moved to the instrument under argon, and the spectrum was collected at 25 °C.

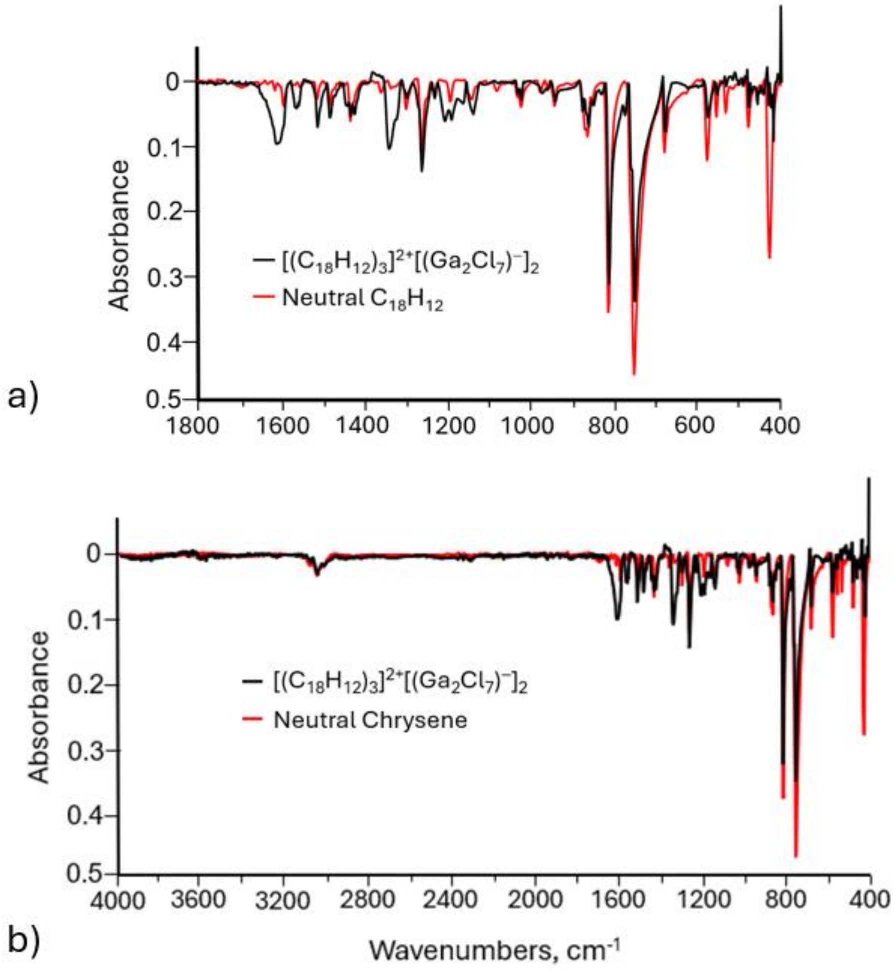

Figure S5. ATR-IR spectrum of purple needles of $[(C_{18}H_{12})_3]^{2+}[(Ga_2Cl_7)^-]_2$ and neat chrysene in the range a) 400-1800 nm and b) 400-4000 nm.

Conductivity Measurements

Sample Preparation: Several ampules containing crystals of $[(C_{18}H_{12})_3]^{2+}[(Ga_2Cl_7)^{-}]_2$ were transferred into the glovebox, where solvent was removed. The crystalline material (25 mg) was washed with hexanes (3.0 mL) and dried *in-vacuo*. The crystals were loaded into a pellet press and compressed for a total of 5 minutes. The current-voltage curve was obtained using a two-point probe method at 25 °C.

X-Ray Powder Diffraction

Sample Preparation: Powder X-ray diffraction was performed on the bulk crystalline sample of $[(C_{18}H_{12})_3]^{2+}[(Ga_2Cl_7)^{-}]_2$. The small, dark purple needles were dried briefly under vacuum and protected under Ar atmosphere, while loading on to the Rigaku XtaLAB Synergy-S diffractometer. The powder diffraction pattern of $[(C_{18}H_{12})_3]^{2+}[(Ga_2Cl_7)^{-}]_2$ was collected at 100 K and had good fit with the calculated Le Bail fit, thus confirming phase purity of the bulk crystalline sample (Figure S6). Additional measurements were performed sequentially at 233.15 K, 253.15 K, 273.15 K, and 293.15 K with a temperature-ramp rate of 0.5 K min^{-1}. After reaching each temperature point, the sample was equilibrated for 60 min before data collection (Figure S7).

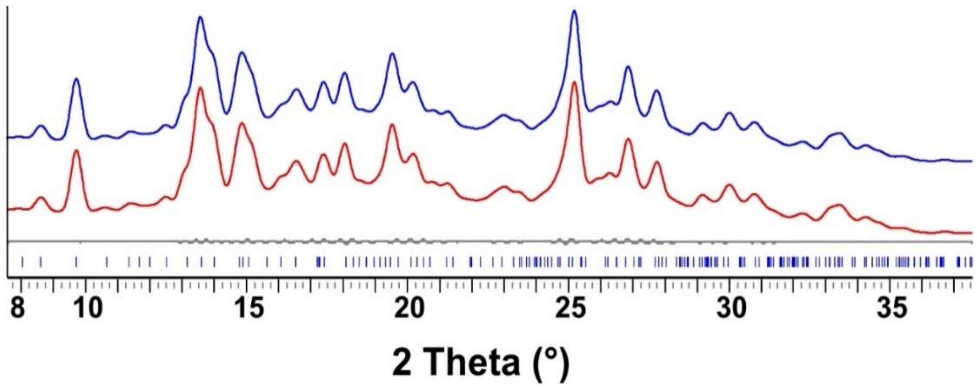

Figure S6. X-ray powder diffraction pattern of $[(C_{18}H_{12})_3]^{2+}[(Ga_2Cl_7)^{-}]_2$ at 100 K and Le Bail fit. The blue and red lines are experimental and calculated patterns, respectively. The grey line is the difference curve with theoretical positions shown at the bottom in black.

Table S1. Single crystal and Le Bail fit data of $[(C_{18}H_{12})_3]^{2+}[(Ga_2Cl_7)^{-}]_2$.

$[(C_{18}H_{12})_3]^{2+}[(Ga_2Cl_7)^{-}]_2$		
	Single crystal data (100 K)	Le Bail fit data (100 K)
Space Group	$P2_1/c$	
a (Å)	9.83944(9)	9.8348(9)
b (Å)	12.15467(13)	12.1554(7)
c (Å)	23.2375(2)	23.2399(9)
α (°)	90.00	90.00
β (°)	97.3301(9)	97.3288(9)
γ (°)	90.00	90.00
V (Å^3)	2756.39(5)	2755.5(3)

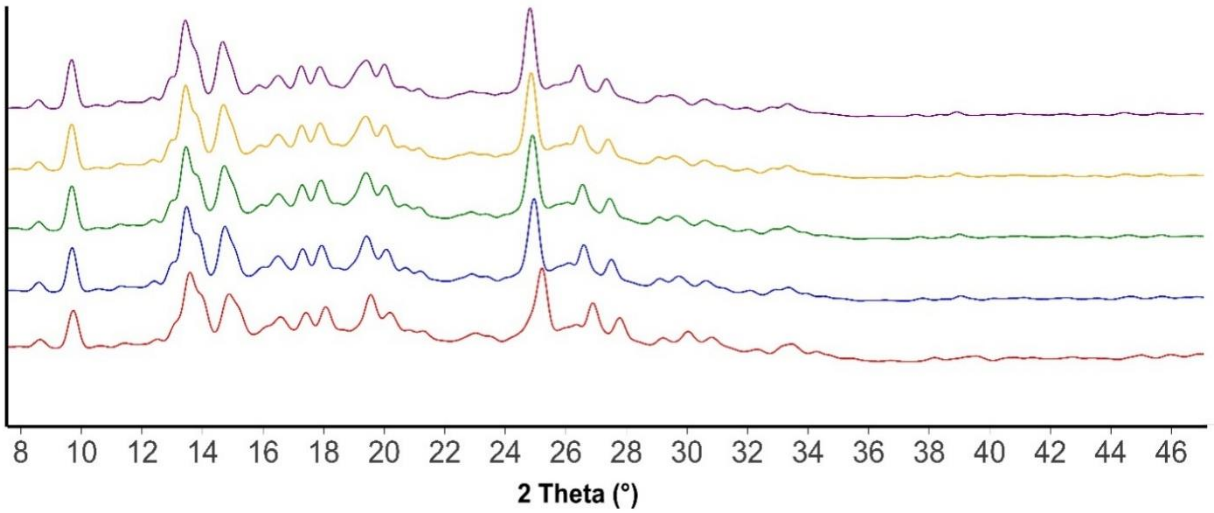

Figure S7. Variable-temperature XRPD patterns of $[(C_{18}H_{12})_3]^{2+}[(Ga_2Cl_7)^{-}]_2$ at 100 K (red), 233.15 K (blue), 253.15 K (green), 273.15 K (yellow), and 293.15 K (purple).

III. Crystal Structure Solution and Refinement

Data collection of $[(C_{18}H_{12})_3]^{2+}[(Ga_2Cl_7)^{-}]_2$ was performed at 99.99(10) K on a Rigaku XtaLAB Synergy-S X-ray diffractometer equipped with a HyPix-6000HE hybrid photon counting (HPC) detector and a microfocus Cu-Kα radiation (λ = 1.54184 Å). Data collection strategy to ensure completeness and desired redundancy were determined using CrysAlisPro.[3] Data processing was performed also using CrysAlisPro. Empirical absorption correction was applied using the SCALE3 ABSPACK scaling algorithm.[4] The structure was solved by SHELXT (version 2018/2)[5] and refined by full-matrix least-squares procedures using the Bruker SHELXTL (version 2019/3)[6] software package through the OLEX2 graphical interface.[7] All non-hydrogen atoms were refined anisotropically. Hydrogen atoms were included in idealized positions for structure factor calculations with $U_{iso}(H) = 1.2\ U_{eq}(C)$. Further crystal and data collection details are listed in Table S2. Solid-state packing diagrams down the crystallographic *a* and *b* directions are shown in Figure S8. ORTEP drawing of the unit cell is shown in Figure S9. The interplanar π-contacts between the chrysene units are shown in Figure S10.

Table S2. Crystallographic data of $[(C_{18}H_{12})_3]^{2+}[(Ga_2Cl_7)^-]_2$.

Compound	$[(C_{18}H_{12})_3]^{2+}[(Ga_2Cl_7)^-]_2$
Empirical formula	$C_{54}H_{36}Cl_{14}Ga_4$
Formula weight	1460.01
Temperature (K)	99.99(10)
Wavelength (Å)	1.54184
Crystal system	Monoclinic
Space group	$P2_1/c$
a (Å)	9.83944(9)
b (Å)	12.15467(13)
c (Å)	23.2375(2)
α (°)	90.00
β (°)	97.3301(9)
γ (°)	90.00
V (Å^3)	2756.39(5)
Z	2
ρ_{calcd} (g·cm^{-3})	1.759
μ (mm^{-1})	8.779
F(000)	1444
Crystal size (mm)	0.02×0.03×0.08
θ range for data collection (°)	3.836–79.781
Reflections collected	36644
Independent reflections	5928 [R_{int} = 0.0347]
Transmission factors (min/max)	0.66933/1.00000
Data/restraints/params.	5928/0/325
$R1$,[a] $wR2$[b] ($I > 2\sigma(I)$)	0.0262, 0.0659
$R1$,[a] $wR2$[b] (all data)	0.0292, 0.0673
Quality-of-fit[c]	1.068

$R_{int} = \Sigma|F_o^2 - \langle F_o^2\rangle|/\Sigma|F_o^2|$

[a]$R1 = \Sigma||F_o|-|F_c||/\Sigma|F_o|$. [b]$wR2 = [\Sigma[w(F_o^2-F_c^2)^2]/\Sigma[w(F_o^2)^2]]$.

[c]Quality-of-fit = $[\Sigma[w(F_o^2-F_c^2)^2]/(N_{obs}-N_{params})]^{½}$, based on all data.

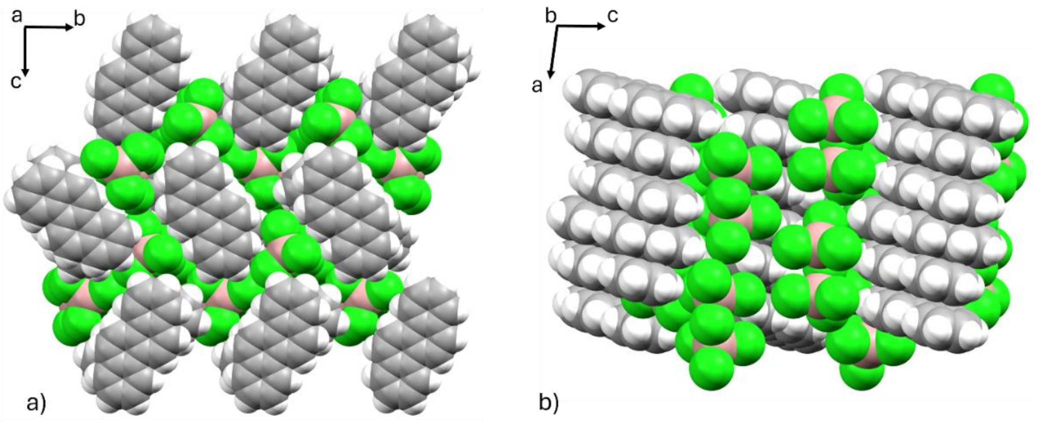

Figure S8. Solid-state packing diagram of $[(C_{18}H_{12})_3]^{2+}[(Ga_2Cl_7)^{-}]_2$ down the a) *a* and b) *b*-axis.

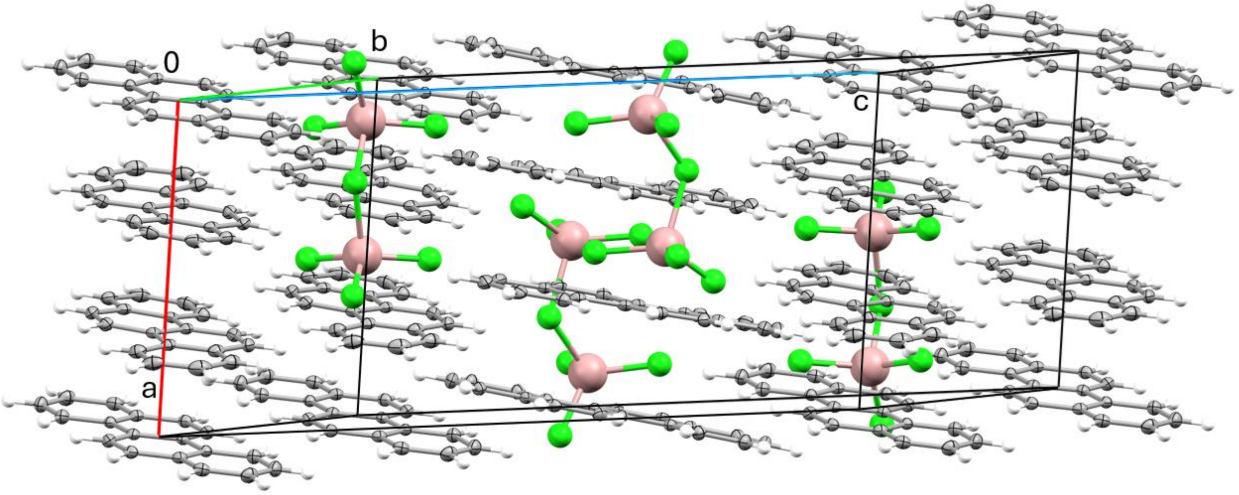

Figure S9. Unit cell of $[(C_{18}H_{12})_3]^{2+}[(Ga_2Cl_7)^{-}]_2$, ORTEP drawing with thermal ellipsoids shown at the 50% probability level.

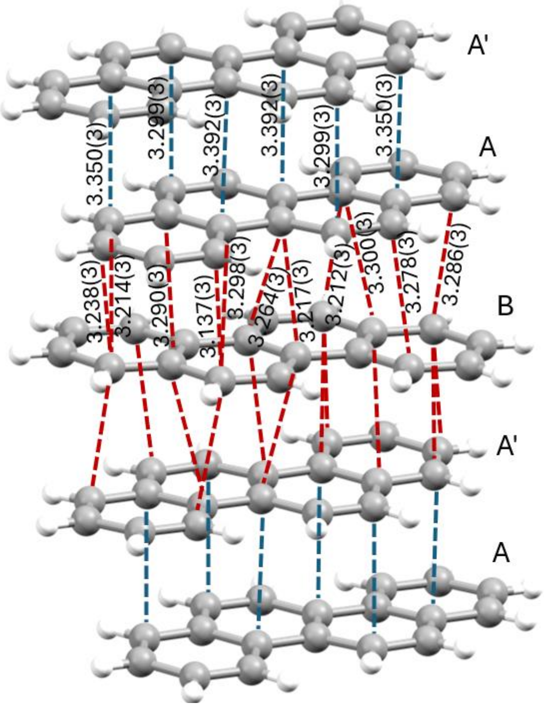

Figure S10. Interplanar contacts (Å) within chrysene columns.

Table S3. C–C bond lengths (Å) in chrysene A/A' and chrysene B with labeling schemes.

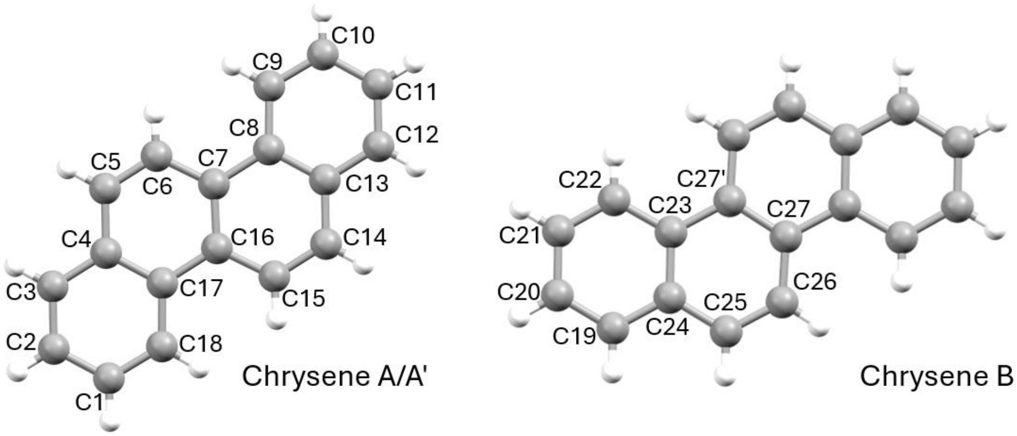

Bond	Chrysene A/A'	Bond	Chrysene A/A'	Bond	Chrysene B
C1–C2	1.392(3)	C9–C10	1.384(3)	C19–C20	1.395(3)
C2–C3	1.388(3)	C10–C11	1.390(3)	C20–C21	1.390(3)
C3–C4	1.405(3)	C11–C12	1.377(3)	C21–C22	1.385(3)
C4–C17	1.420(3)	C12–C13	1.409(3)	C22–C23	1.413(3)
C4–C5	1.431(3)	C13–C14	1.431(3)	C23–C24	1.418(3)
C5–C6	1.360(3)	C14–C15	1.361(3)	C23–C27'	1.443(3)
C6–C7	1.424(3)	C15–C16	1.426(3)	C24–C25	1.425(3)
C7–C16	1.431(3)	C16–C17	1.442(3)	C24–C19	1.402(3)
C7–C8	1.446(3)	C17–C18	1.412(3)	C25–C26	1.369(3)
C8–C13	1.419(3)	C18–C1	1.379(3)	C26–C27	1.415(3)
C8–C9	1.414(3)			C27–C27'	1.443(3)

IV. Computational Details

All calculations were carried out using density functional theory (DFT) at the (U)M052X[8]/6-311G(d) level. The unrestricted (U) formalism was employed for open-shell systems. Frequency calculations were performed at the optimized geometries to ensure that the structures correspond to true minima (i.e., no imaginary frequencies). All computations were conducted using the Gaussian 16 software package (Revision A.03).[9]

To determine the average interplanar distance, we considered only carbon–carbon (C⋯C) contacts shorter than 3.40 Å. The average was calculated using the following equation, for m, see Figure 6c:

$$d_{av}(C \cdots C) = \frac{1}{m}\sum_{(i,j)=1}^{m} d\left(C_{A,i} \cdots C_{B,j}\right) \quad where\ d < 3.40Å \qquad \text{Equation 3}$$

Here C_A and C_B refer to the carbon atoms from two adjacent monomers that exhibit the shortest interatomic distances, and *(i,j)* refers to one of the *m* close intermolecular contacts.

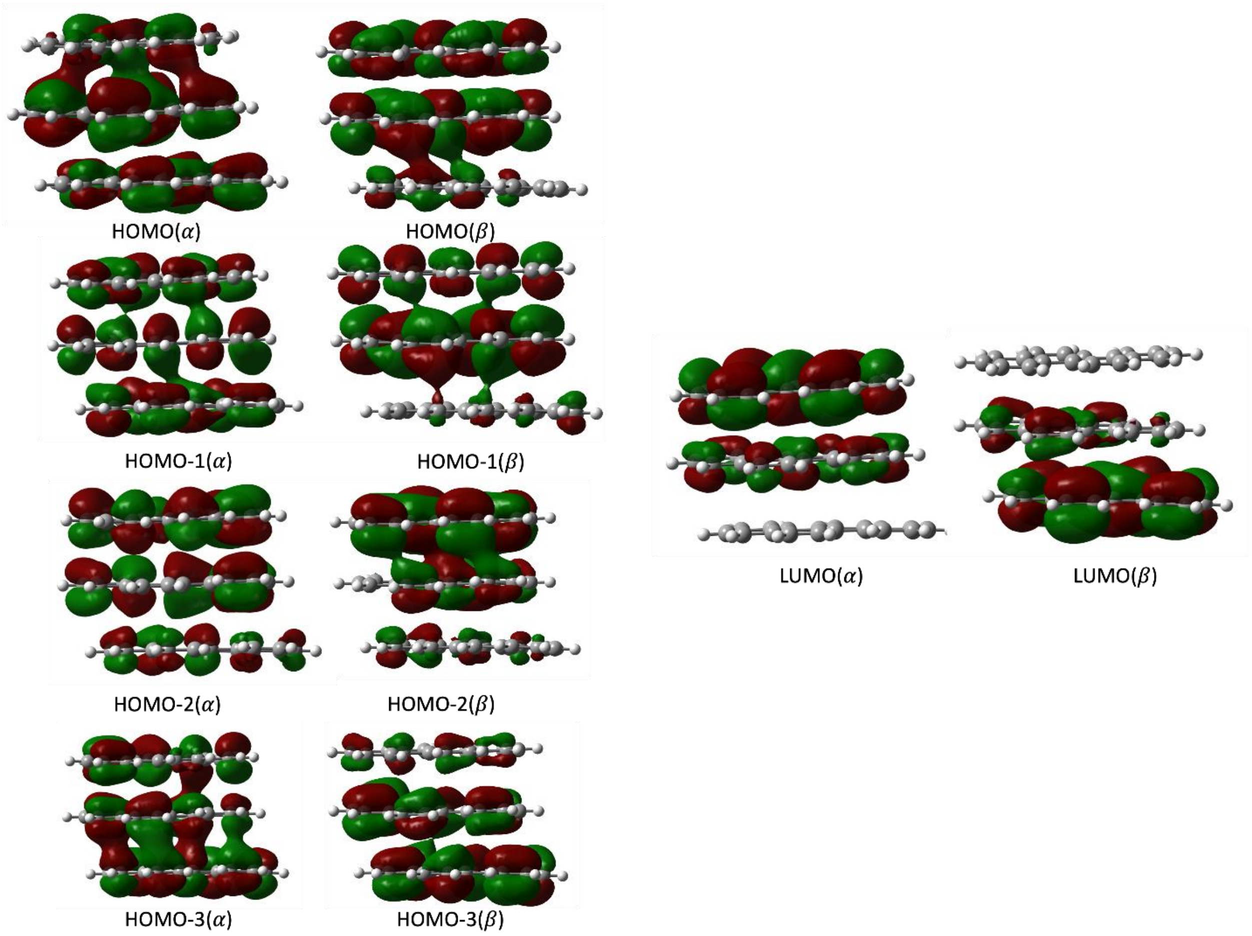

Figure S11. Occupied and unoccupied valence orbitals of $[(C_{18}H_{12})_3]^{2+}$.

Optimized Coordinates:

Chrysene Trimer +2-charged open-shell singlet

C	-2.85888200	2.02943100	-1.77365600
H	-3.28766600	2.38924600	-2.69923400
C	-3.47426600	2.34307800	-0.55353900
H	-4.37757500	2.93678100	-0.53809500
C	-2.91498200	1.89589400	0.62034800
H	-3.36837800	2.14358400	1.57192500
C	-1.73933000	1.11919700	0.61138300
C	-1.14848200	0.67959300	1.83130400
H	-1.62759300	0.94745400	2.76452900
C	0.00449300	-0.04186900	1.83569700
H	0.41987900	-0.34676100	2.78371500
C	0.65018800	-0.41627700	0.62131000
C	1.87427400	-1.19015000	0.62603100
C	2.54818600	-1.54972200	1.81022200
H	2.16567700	-1.25048100	2.77396800
C	3.71230000	-2.29120400	1.77421500
H	4.20578600	-2.55540800	2.69966300
C	4.25436000	-2.70890400	0.55293000
H	5.16302600	-3.29454500	0.53368400
C	3.62442200	-2.36192400	-0.62134700
H	4.03759800	-2.66530500	-1.57481500
C	2.44017200	-1.60006300	-0.60562900
C	1.80038400	-1.23376500	-1.82321100
H	2.23528100	-1.56431000	-2.75814100
C	0.66914700	-0.47773900	-1.82250300
H	0.21965900	-0.22297500	-2.76962200
C	0.07093500	-0.03410600	-0.60771300
C	-1.12675900	0.77743200	-0.61766500
C	-1.71099000	1.26698000	-1.80569600
H	-1.26573400	1.05556900	-2.76595200
C	-1.07237100	4.70584900	0.17022500
H	-1.62047100	5.12438700	-0.66313600
C	-1.47868800	4.95083900	1.46961800
H	-2.34306500	5.57002500	1.66277400
C	-0.75787800	4.40186000	2.52764600
H	-1.06445800	4.59481900	3.54614700
C	0.36676900	3.62586800	2.29071000
H	0.89715200	3.22975100	3.14243300
C	0.81090800	3.37696600	0.98445200
C	0.06267500	3.92288300	-0.08449400
C	0.48184100	3.68037300	-1.42127500
H	-0.08578800	4.12247500	-2.22908000
C	1.58928500	2.92271900	-1.70295500
C	0.67588300	-4.39140600	1.93581000
H	1.09536500	-4.69153600	2.88587900
C	1.29255100	-4.79488400	0.74940800

H	2.18624300	-5.40068900	0.77939200
C	0.74502100	-4.41563200	-0.45522700
H	1.20319000	-4.71902900	-1.38740200
C	-0.42946300	-3.63761700	-0.49362500
C	-0.99619700	-3.25791900	-1.73046700
H	-0.51828100	-3.58685500	-2.64371800
C	-2.15291900	-2.50571200	-1.80460200
H	-2.54381000	-2.26913200	-2.78146800
C	-2.82005400	-2.08128500	-0.65180000
C	-4.05126300	-1.30428900	-0.72581900
C	-4.61873900	-0.88113700	-1.93026900
H	-4.14991900	-1.10421100	-2.87601200
C	-5.81156100	-0.16993200	-1.94700500
H	-6.23431000	0.12880700	-2.89603200
C	-6.47541800	0.15028800	-0.76244300
H	-7.41008500	0.69138200	-0.79144700
C	-5.93209200	-0.24442800	0.44133300
H	-6.43334100	-0.01511200	1.37238900
C	-4.72382200	-0.96772000	0.47512400
C	-4.17087100	-1.37366300	1.70893000
H	-4.69605300	-1.12569100	2.62211200
C	-2.98687800	-2.08135600	1.77917400
H	-2.62621200	-2.36876100	2.75356500
C	-2.28989100	-2.45604800	0.62677400
C	-1.06334600	-3.23555800	0.70657300
C	-0.48114500	-3.62735600	1.91745800
H	-0.92387400	-3.35612900	2.86320900
H	2.59230900	2.30856400	2.74797700
C	1.99965800	2.60032500	0.69059000
H	1.86967400	2.79053500	-2.73542300
C	2.37092000	2.35964000	-0.67782300
C	5.52447400	0.37906100	-0.15839900
H	6.12539000	0.03953300	0.67454700
C	5.91286200	0.10679800	-1.45854000
H	6.82420100	-0.44094700	-1.65165900
C	5.12445700	0.55315600	-2.51616800
H	5.42439700	0.35061000	-3.53475900
C	3.96445900	1.27587300	-2.27845300
H	3.39158300	1.60782000	-3.13007800
C	3.55762900	1.58098100	-0.97190200
C	4.35511600	1.10940400	0.09710200
C	3.96400200	1.39356000	1.43366400
H	4.59436000	1.04736200	2.24175100
C	2.82655500	2.10681300	1.71526400

Chrysene Trimer +2-charged triplet

C	-2.79617900	2.06848100	-1.76961900
H	-3.21744500	2.44793100	-2.69087500
C	-3.41395400	2.36836500	-0.54648800
H	-4.31151000	2.97068800	-0.52533100
C	-2.86517300	1.89568100	0.62208600

H	-3.32003500	2.13285200	1.57563200
C	-1.69653200	1.10870800	0.60573600
C	-1.11333800	0.64626800	1.82200900
H	-1.59433300	0.90425200	2.75712800
C	0.03041300	-0.08602400	1.81912200
H	0.43941700	-0.41036200	2.76343500
C	0.67762200	-0.44940100	0.59936400
C	1.88914800	-1.24076600	0.59817600
C	2.55122300	-1.63135400	1.78120300
H	2.16764300	-1.34163900	2.74746400
C	3.70029100	-2.39244600	1.73929700
H	4.18432000	-2.68085400	2.66253800
C	4.24234400	-2.79984100	0.51259800
H	5.14061700	-3.40122400	0.48924000
C	3.62723700	-2.42198600	-0.65845900
H	4.04157700	-2.71566200	-1.61449200
C	2.45566500	-1.63937900	-0.63721700
C	1.83031000	-1.24066500	-1.85227900
H	2.26831700	-1.55856600	-2.79016800
C	0.71078900	-0.46912700	-1.84459400
H	0.27211700	-0.18841400	-2.78963100
C	0.10939800	-0.04045400	-0.62403100
C	-1.08108700	0.78137600	-0.62604500
C	-1.65704900	1.29483700	-1.80914000
H	-1.20980600	1.09377200	-2.77077300
C	-0.98051500	4.70002100	0.17234700
H	-1.51299900	5.12893000	-0.66583300
C	-1.39873500	4.94546800	1.46789600
H	-2.25734700	5.57489600	1.65344300
C	-0.69747800	4.38327000	2.53192000
H	-1.01316400	4.57666200	3.54754200
C	0.41948100	3.59301400	2.30525000
H	0.93487600	3.18751700	3.16172400
C	0.87539800	3.34288500	1.00350000
C	0.14729100	3.90346000	-0.07165100
C	0.58055100	3.66269400	-1.40406300
H	0.02634800	4.11288600	-2.21671000
C	1.68582800	2.89747700	-1.67635100
C	0.53702600	-4.40760800	1.90119900
H	0.94339600	-4.73631400	2.84747100
C	1.16598300	-4.77835900	0.71263000
H	2.05748000	-5.38764000	0.73502500
C	0.63517400	-4.35926200	-0.48845900
H	1.10482800	-4.63601800	-1.42306300
C	-0.53429900	-3.57616200	-0.51768400
C	-1.08276900	-3.15583000	-1.75053600
H	-0.59403800	-3.45976600	-2.66653900
C	-2.23268500	-2.39194700	-1.81759000
H	-2.60714200	-2.12109100	-2.79188700
C	-2.91190400	-1.99709300	-0.66256000
C	-4.13491600	-1.20720100	-0.72977800

C	-4.68020600	-0.74051900	-1.92839700
H	-4.19872500	-0.93672200	-2.87367900
C	-5.86532300	-0.01649700	-1.93935800
H	-6.27058800	0.31792000	-2.88408600
C	-6.54345300	0.27136700	-0.75488700
H	-7.47204900	0.82302200	-0.77967500
C	-6.02243700	-0.16832300	0.44362100
H	-6.53558800	0.03575000	1.37402000
C	-4.82190300	-0.90298700	0.47105500
C	-4.29085300	-1.35429400	1.69987300
H	-4.82788200	-1.13056600	2.61238300
C	-3.11486900	-2.07541100	1.76597800
H	-2.77140100	-2.39648200	2.73606900
C	-2.40260900	-2.41701800	0.61359900
C	-1.18149700	-3.20637100	0.68535500
C	-0.61631200	-3.63541800	1.89067100
H	-1.06780600	-3.38704600	2.83854500
H	2.60654300	2.21968000	2.78299400
C	2.05781900	2.55238100	0.72004600
H	1.97672200	2.76721000	-2.70612400
C	2.44911800	2.32177900	-0.64557500
C	5.57369400	0.30288800	-0.09598400
H	6.15343700	-0.05686200	0.74342700
C	5.98666100	0.04970200	-1.39201700
H	6.89633100	-0.50393500	-1.57585600
C	5.22512300	0.52270400	-2.45795800
H	5.54503800	0.33548800	-3.47338200
C	4.06712100	1.25213600	-2.23228700
H	3.51630400	1.60522500	-3.08994000
C	3.63489400	1.53693900	-0.92947400
C	4.40589500	1.03987200	0.14715900
C	3.99075600	1.30693200	1.47999300
H	4.59948400	0.93787700	2.29451700
C	2.85833300	2.03247300	1.75170600

Chrysene Trimer +2-charged singlet

C	-3.12994400	1.77123200	-1.75953400
H	-3.59047600	2.06537300	-2.69237400
C	-3.76773000	2.05003200	-0.55222600
H	-4.72481500	2.55142200	-0.54402900
C	-3.16142800	1.68498300	0.63549800
H	-3.63972300	1.89988500	1.58221200
C	-1.92125900	1.02381400	0.63439700
C	-1.29559500	0.66206500	1.85500500
H	-1.80248800	0.88443000	2.78434000
C	-0.05441800	0.06452600	1.87851500
H	0.38054000	-0.17947100	2.83508300
C	0.62097600	-0.25664400	0.69137800
C	1.92884700	-0.88650800	0.69979000
C	2.66957700	-1.07390800	1.86937800
H	2.29085100	-0.74248600	2.82381300

C	3.92298900	-1.68301900	1.83902800
H	4.46986100	-1.81550000	2.76229700
C	4.46511300	-2.12690500	0.64147000
H	5.43194100	-2.60978500	0.62362400
C	3.75419800	-1.94042900	-0.53705000
H	4.16431700	-2.27518400	-1.48053300
C	2.49936000	-1.31607600	-0.52237600
C	1.79042300	-1.10358100	-1.73461000
H	2.21645700	-1.47351500	-2.65796400
C	0.57963700	-0.45601700	-1.75180300
H	0.08063800	-0.32816900	-2.69889500
C	-0.01950200	0.01030600	-0.56808800
C	-1.28372500	0.71335900	-0.58974200
C	-1.90403000	1.12139100	-1.77932500
H	-1.44018800	0.93979900	-2.73651100
C	-1.65403700	4.52470700	0.31488200
H	-2.27913700	4.92876300	-0.47040600
C	-2.03366700	4.64613000	1.64009600
H	-2.95206400	5.15435500	1.89831500
C	-1.21725100	4.12029600	2.64103400
H	-1.50605500	4.21510200	3.67860900
C	-0.02154700	3.50045800	2.31806600
H	0.58894200	3.11809100	3.12168100
C	0.40060900	3.38808900	0.98040200
C	-0.45102000	3.89132600	-0.03170200
C	-0.07574500	3.74566900	-1.39942800
H	-0.70475800	4.18823300	-2.16044200
C	1.05632700	3.07495900	-1.75163800
C	1.30112100	-4.17288600	2.13177500
H	1.74354800	-4.35110900	3.10220300
C	1.94477800	-4.64666700	0.97757900
H	2.87907800	-5.18343200	1.06055500
C	1.36681400	-4.44083400	-0.24869500
H	1.83827400	-4.81440400	-1.14884700
C	0.13415200	-3.75828600	-0.35876500
C	-0.46170700	-3.54503200	-1.62614500
H	0.03125100	-3.93896900	-2.50586900
C	-1.66229100	-2.89638100	-1.75377500
H	-2.08648500	-2.79366000	-2.74048000
C	-2.35854400	-2.41004300	-0.62545600
C	-3.64139600	-1.73697100	-0.75612300
C	-4.23522700	-1.44973800	-1.99330300
H	-3.74940500	-1.72284800	-2.91745500
C	-5.47417500	-0.83265800	-2.06854000
H	-5.91606900	-0.64884700	-3.03803700
C	-6.16413700	-0.46569700	-0.91095200
H	-7.13777400	-0.00213500	-0.98088700
C	-5.59648200	-0.71764600	0.31895400
H	-6.11989600	-0.45429600	1.22911600
C	-4.34030100	-1.35152600	0.41483000
C	-3.76490400	-1.61383000	1.68175800

H	-4.31909200	-1.34360000	2.57188200
C	-2.53729200	-2.20755400	1.79904400
H	-2.15920800	-2.40377400	2.78961900
C	-1.80464700	-2.62331600	0.66249200
C	-0.52613100	-3.29036300	0.80266500
C	0.09150900	-3.51727800	2.04809900
H	-0.37803800	-3.19840800	2.96584600
H	2.47252900	2.69326800	2.61383800
C	1.64937600	2.76657200	0.60894800
H	1.31228500	3.00420600	-2.79714300
C	1.93272800	2.54268300	-0.76987500
C	5.27399600	0.85929000	-0.48373200
H	6.00043600	0.64499000	0.28903100
C	5.52998200	0.50387600	-1.79815900
H	6.46034300	0.01897400	-2.05847500
C	4.58666600	0.78818000	-2.78372400
H	4.78140600	0.51718000	-3.81208500
C	3.40889600	1.44258400	-2.45939200
H	2.70992900	1.65824600	-3.25271700
C	3.13790900	1.84080300	-1.13685800
C	4.08381400	1.51533800	-0.13543300
C	3.81218800	1.85584000	1.22149300
H	4.56945700	1.65937000	1.96892500
C	2.63007900	2.43426700	1.57856500

Chrysene Trimer +1-charged doublet

C	-2.86882800	1.93116100	-1.81283900
H	-3.31249400	2.27075000	-2.73855900
C	-3.49300300	2.21557700	-0.59911500
H	-4.42197200	2.76688700	-0.57907600
C	-2.92017400	1.77279600	0.57731500
H	-3.39134200	1.98304900	1.52839300
C	-1.72282300	1.04230100	0.56190900
C	-1.12909900	0.60243300	1.77774000
H	-1.62893100	0.83543800	2.70818100
C	0.04657700	-0.09444900	1.78677100
H	0.45244200	-0.40891400	2.73498100
C	0.71075700	-0.42972000	0.58576000
C	1.95107200	-1.17826900	0.58912200
C	2.60745400	-1.55963200	1.76974700
H	2.20174200	-1.29799600	2.73428100
C	3.78850300	-2.28063600	1.73210500
H	4.26831500	-2.56470300	2.65865100
C	4.35612000	-2.64924600	0.51286000
H	5.27646900	-3.21612200	0.48952600
C	3.73767800	-2.27700700	-0.66439000
H	4.17087600	-2.54333700	-1.61945200
C	2.54288700	-1.54234900	-0.64232200
C	1.91135100	-1.15887800	-1.85889000
H	2.36517000	-1.46066500	-2.79378100
C	0.75456400	-0.43541600	-1.86230100

H	0.30788200	-0.18347900	-2.81050500
C	0.13003200	-0.03633200	-0.65783600
C	-1.09336000	0.73480300	-0.66590300
C	-1.68978100	1.20702700	-1.84741100
H	-1.23922400	1.01302000	-2.80820900
C	-1.17449000	4.57954200	0.18463800
H	-1.72037600	5.00340700	-0.64801900
C	-1.63071500	4.74470100	1.47384300
H	-2.53303700	5.30889200	1.66484400
C	-0.91723000	4.17860900	2.53608100
H	-1.27037800	4.30476100	3.55040600
C	0.24425400	3.47329500	2.30197000
H	0.76764300	3.05636900	3.14825600
C	0.74566000	3.30437800	0.99452600
C	0.00396000	3.85885200	-0.07476500
C	0.46264100	3.67546100	-1.41219200
H	-0.10010400	4.12735800	-2.21852800
C	1.58278000	2.95565700	-1.67761500
C	0.68191600	-4.29953200	1.93062500
H	1.12007100	-4.58121300	2.87868700
C	1.29706400	-4.70364300	0.73242300
H	2.20740700	-5.28593300	0.76030600
C	0.72957300	-4.35685300	-0.46450300
H	1.18459100	-4.66344700	-1.39813000
C	-0.46367600	-3.60228900	-0.50937400
C	-1.05632700	-3.25407800	-1.75539500
H	-0.57978800	-3.59098600	-2.66738400
C	-2.20212400	-2.52893600	-1.81152100
H	-2.62239500	-2.30194300	-2.77881300
C	-2.86222800	-2.08665500	-0.62569100
C	-4.08116400	-1.30642400	-0.69137000
C	-4.68521100	-0.92154000	-1.90747700
H	-4.25008300	-1.21176500	-2.85120000
C	-5.84285700	-0.17975300	-1.92710000
H	-6.28895500	0.08920800	-2.87490800
C	-6.45751600	0.22028600	-0.72928800
H	-7.37346500	0.79432300	-0.75538300
C	-5.89632900	-0.13963400	0.46925400
H	-6.36396200	0.14570400	1.40327300
C	-4.71009100	-0.90414400	0.51106400
C	-4.13485500	-1.28000600	1.75584600
H	-4.63659900	-0.98710000	2.66972400
C	-2.98557500	-2.00135200	1.80790800
H	-2.59323700	-2.27399100	2.77506900
C	-2.31383700	-2.42443100	0.62223900
C	-1.08885300	-3.19307000	0.69137300
C	-0.47900200	-3.56562000	1.91076400
H	-0.92581000	-3.29078200	2.85383900
H	2.54448500	2.30285500	2.78781100
C	1.96683500	2.58613600	0.71081600
H	1.90037900	2.85308100	-2.70319000

C	2.36218200	2.38181600	-0.63165800
C	5.55012500	0.46427700	-0.10385300
H	6.15460600	0.13595000	0.73161900
C	5.93745500	0.18679100	-1.39600100
H	6.85472100	-0.35288600	-1.58669400
C	5.13597200	0.61002400	-2.46255600
H	5.43473900	0.39492800	-3.47938800
C	3.97481400	1.31543200	-2.22845100
H	3.38802200	1.62928100	-3.07766100
C	3.56328900	1.63065600	-0.91661200
C	4.36639200	1.17645800	0.15560600
C	3.96091700	1.44682400	1.49554900
H	4.59812400	1.11611500	2.30532800
C	2.80539900	2.10858100	1.75952700

Chrysene Trimer neutral closed-shell singlet

C	-2.77135500	1.55607900	-1.74388400
H	-3.28388200	1.84332100	-2.65278900
C	-3.35101200	1.84496800	-0.49782600
H	-4.30501500	2.35128100	-0.44657500
C	-2.70445000	1.46608100	0.64877800
H	-3.13536800	1.67716900	1.61922900
C	-1.46633400	0.79354200	0.59198000
C	-0.80225700	0.39455700	1.78753700
H	-1.27127400	0.62379900	2.73643300
C	0.38373300	-0.25773500	1.74743000
H	0.85119500	-0.54200900	2.67740000
C	1.02135800	-0.57346800	0.50739500
C	2.28358900	-1.27940300	0.47073700
C	2.96977600	-1.69610700	1.63384100
H	2.56332600	-1.48285500	2.61049800
C	4.15349400	-2.38799600	1.55718900
H	4.65114300	-2.69912100	2.46651800
C	4.71928000	-2.70150600	0.30893100
H	5.65144900	-3.24832400	0.25696100
C	4.08706000	-2.29464000	-0.83644900
H	4.51410100	-2.51039900	-1.80827900
C	2.87105400	-1.58142700	-0.77893300
C	2.21517400	-1.16874300	-1.97261500
H	2.68358200	-1.39140300	-2.92352100
C	1.03336900	-0.50701400	-1.92891000
H	0.56922300	-0.21467400	-2.85808500
C	0.39673600	-0.19354300	-0.69002500
C	-0.87289300	0.50257900	-0.65633600
C	-1.56467900	0.90435900	-1.82080800
H	-1.15256600	0.70211700	-2.79764100
C	-1.67660100	4.82282700	-0.27327000
H	-2.15679500	5.13799800	-1.19125400
C	-2.24895100	5.09311600	0.94133900
H	-3.18533700	5.63138300	0.99973500
C	-1.61226200	4.65625200	2.11572600

H	-2.06023900	4.86330100	3.07869000
C	-0.42234500	3.97366600	2.05447200
H	0.03514000	3.65798900	2.97946500
C	0.19953300	3.69042500	0.81692000
C	-0.45428800	4.12336200	-0.35901400
C	0.13557700	3.84516700	-1.62443900
H	-0.38423600	4.16659700	-2.51818700
C	1.31666700	3.18865400	-1.71852800
C	1.01143900	-4.44104500	1.68809000
H	1.51203100	-4.73988900	2.59949700
C	1.60732500	-4.71581300	0.44604800
H	2.56771400	-5.21016600	0.40193900
C	0.97266500	-4.32597200	-0.70350400
H	1.42192500	-4.51416400	-1.67063900
C	-0.26914900	-3.65822300	-0.65510700
C	-0.92525600	-3.25674700	-1.85399100
H	-0.44744500	-3.47503900	-2.80074300
C	-2.11515400	-2.61099700	-1.82082400
H	-2.57403200	-2.32282800	-2.75362900
C	-2.75967200	-2.29766300	-0.58422000
C	-4.02663700	-1.59837600	-0.55516000
C	-4.71270100	-1.19614400	-1.72315100
H	-4.30104800	-1.40984200	-2.69727100
C	-5.90697200	-0.52248800	-1.65525800
H	-6.40826700	-0.22840700	-2.56780300
C	-6.48204300	-0.21222400	-0.41096300
H	-7.42367100	0.31836500	-0.36553000
C	-5.84445300	-0.59503600	0.74016500
H	-6.27440800	-0.37019100	1.70848000
C	-4.61805500	-1.29047400	0.69127500
C	-3.95679200	-1.68125900	1.89025700
H	-4.41889800	-1.43965500	2.83951200
C	-2.77444000	-2.34108100	1.85311600
H	-2.30560900	-2.61382000	2.78555300
C	-2.13883100	-2.67098600	0.61707600
C	-0.87223700	-3.37186700	0.59020200
C	-0.19311700	-3.78565900	1.75852800
H	-0.61401900	-3.59100800	2.73300100
H	1.73686700	2.70140100	2.84721300
C	1.46015600	2.98838900	0.70723600
H	1.72531100	2.99755400	-2.69854800
C	2.01928100	2.74539900	-0.55659300
C	5.17146300	0.93753600	0.42533800
H	5.64102800	0.60067400	1.34056300
C	5.75529700	0.69164900	-0.78934300
H	6.69554000	0.16008500	-0.84651100
C	5.11867800	1.12596700	-1.96387200
H	5.57678800	0.93702900	-2.92579300
C	3.91600700	1.78581900	-1.90279700
H	3.45635300	2.09848300	-2.82761800
C	3.28698000	2.05356200	-0.66610100

C	3.94059200	1.62053600	0.50942500
C	3.33934800	1.87774600	1.77487000
H	3.84800600	1.53411400	2.66704600
C	2.15753500	2.53159000	1.86846700

V. References

(1) Kozhemyakina, N. V.; Nuss, J.; Jansen, M. Z. Demonstration of the "Break-and-Seal" Approach to Fullerides of Complex Cations at the Example of $KC_{60}(THF)_5 \cdot 2THF$. *Anorg. Allg. Chem.* **2009**, *635*, 1355-1361.
(2) Stawski, W.; Zhu, Y.; Wei, Z.; Petrukhina, M. A.; Anderson, H. L. Crystallographic Evidence of Global Aromaticity in the Di-Anion and Tetra-Anion of a Cyclophane Hydrocarbon. *Chem. Sci.* **2023**, *14*, 14109-14114.
(3) Rigaku Corporation. Rigaku Oxford Diffraction, CrysAlisPro Software System, Version 1.171.43.134a, **2024**.
(4) Rigaku Oxford Diffraction. SCALE3 ABSPACK; A Rigaku Oxford Diffraction Program (1.0.11,Gui:1.0.7) (C), **2005**.
(5) Sheldrick, G. M. SHELXT – Integrated Space-Group and Crystal-Structure Determination. *Acta Crystallogr.* **2015**, *A71*, 3-8.
(6) Sheldrick, G. M. Crystal Structure Refinement with SHELXL. *Acta Crystallogr.* **2015**, *C71*, 3-8.
(7) Dolomanov, O. V.; Bourhis, L. J.; Gildea, R. J.; Howard, J. A. K.; Puschmann, H. OLEX2: A Complete Structure Solution, Refinement and Analysis Program. *J. Appl. Crystallogr.* **2009**, *42*, 339-341.
(8) Zhao, Y.; Schultz, N. E.; Truhlar, D. G. Design of Density Functionals by Combining the Method of Constraint Satisfaction with Parametrization for Thermochemistry, Thermochemical Kinetics, and Noncovalent Interactions. *J. Chem. Theory Comput.* **2006**, *2*, 364-382.
(9) Frisch, M.; Trucks, G.; Schlegel, H.; Scuseria, G.; Robb, M.; Cheeseman, J.; Scalmani, G.; Barone, V.; Petersson, G.; Nakatsuji, H. *Gaussian 16*, Revision A. 03, Gaussian. Inc.: Wallingford CT, 2016.
